# Correction: Gaussian decomposition of high-resolution melt curve derivatives for measuring genome-editing efficiency

**DOI:** 10.1371/journal.pone.0194470

**Published:** 2018-03-12

**Authors:** Michail Zaboikin, Carl Freter, Narasimhachar Srinivasakumar

There is an error in the sentence preceding the fourth equation in the Materials and Methods section, under the subsection titled “Processing melt curve data”. The correct sentence and equation are:

Efficiency of dsDNA detection at temperature *x*_*i*_

$$E\left(x_{i}\right)=\frac{F_{prem}\left(x_{i}\right)}{F_{prem}\left(x_{i}=71{}^{\circ}\text{C}\right)}$$

There is an error in the seventh equation in the Materials and Methods section. Please view the complete, correct equation here:

$$\begin{array}{l} \begin{array}{c} -\frac{\text{d}}{\text{d}T}\left(nFcRFU\right)\equiv \frac{\text{d}}{\text{d}x}\left(1-nFcRFU\left(x_{i}\right)\right)=-\frac{\text{d}}{\text{d}x}\left(nFcRFU\left(x_{i}\right)\right)=\\ \frac{-\left(nFcRFU\left(x_{i+1}\right)-nFcRFU\left(x_{i}\right)\right)}{x_{i+1}-x_{i}}=\frac{-\left(nFcRFU\left(x_{i+1}\right)-nFcRFU\left(x_{i}\right)\right)}{0.2} \end{array} \end{array}$$

There is an error in the fifth sentence of the second paragraph of the Results section under the subsection titled “Correction of RFU for temperature-dependent quenching of dsDNA-bound fluorophore”. The sentence “Two-Gaussian decomposition is superior to one-Gaussian modeling of derivative melt curves of unmodified target sites” should appear as a subheading.

## References

[1] Zaboikin M, Freter C, Srinivasakumar N (2018) Gaussian decomposition of high-resolution melt curve derivatives for measuring genome-editing efficiency. PLoS ONE 13(1): e0190192 https://doi.org/10.1371/journal.pone.0190192 29300734 10.1371/journal.pone.0190192 PMC5754072

